# Use of Human Umbilical Cord and Its Byproducts in Tissue Regeneration

**DOI:** 10.3389/fbioe.2020.00117

**Published:** 2020-03-10

**Authors:** Francesca Velarde, Verónica Castañeda, Emilia Morales, Mayra Ortega, Edwin Ocaña, Jose Álvarez-Barreto, Michelle Grunauer, Luis Eguiguren, Andrés Caicedo

**Affiliations:** ^1^Colegio de Ciencias de la Salud, Escuela de Medicina, Universidad San Francisco de Quito, Quito, Ecuador; ^2^Instituto de Investigaciones en Biomedicina, Universidad San Francisco de Quito, Quito, Ecuador; ^3^Colegio de Ciencias Biológicas y Ambientales, Escuela de Biotecnología, Universidad San Francisco de Quito, Quito, Ecuador; ^4^Hospital Carlos Andrade Marín, Quito, Ecuador; ^5^Instituto para el Desarrollo de Energías y Materiales Alternativos (IDEMA), Colegio de Ciencias e Ingenierías (Politécnico), Universidad San Francisco de Quito, Quito, Ecuador; ^6^Unidad de Cuidados Intensivos Pediátricos, Hospital de los Valles, Quito, Ecuador; ^7^Sistemas Médicos, SIME, Universidad San Francisco de Quito, Quito, Ecuador

**Keywords:** tissue regeneration, umbilical cord, mesenchymal stem cells, cryopreserved allograft, umbilical cord stem cells, umbilical cord extracts, gastroschisis, regenerative medicine

## Abstract

The fresh or cryopreserved human umbilical cord (HUC) and its byproducts, such as cells and extracts, have different uses in tissue regeneration. Defining what HUC byproduct is more effective in a particular application is a challenge. Furthermore, the methods of isolation, culture and preservation, may affect cell viability and regenerative properties. In this article, we review the HUC and its byproducts’ applications in research and clinical practice. We present our results of successful use of HUC as a patch to treat gastroschisis and its potential to be applied in other conditions. Our *in vitro* results show an increase in proliferation and migration of human fibroblasts by using an acellular HUC extract. Our goal is to promote standardization of procedures and point out that applications of HUC and its byproducts, as well as the resulting advances in regenerative medicine, will depend on rigorous quality control and on more research in this area.

## Introduction

The human umbilical cord (HUC) has become a tissue of great and increasing interest in regenerative medicine. HUC byproducts, such as cells and extracts, have been studied *in vitro* and *in vivo* with encouraging results in tissue outcomes (Iftimia-Mander et al., 2013; Papanna et al., 2015b; Wu et al., 2018). HUC Mesenchymal Stem Cells (MSCs), especially those isolated from the Wharton’s Jelly (WJ), are already being used in clinical trials which have reported safety and efficacy in wound healing (Couto et al., 2019). HUC patches have been used in clinical practice with proven reparative effects for gastroschisis, and in preclinical assays for spina bifida and foot ulcers (Hernández Siverio et al., 2007; Papanna et al., 2015b; Caputo et al., 2016). However, much of the information from research and clinical uses of HUC and its byproducts is poorly defined, leading to data misinterpretation and misuse. For these reasons, it is necessary to review the scientific basis for their applications and translation from research to clinic. Information about the possible benefits of HUC use and limitations in autologous and allogeneic therapy might help donors to consent and health providers to give appropriate advice regarding its collection and banking (Roura et al., 2015).

In this article, we review previous relevant work regarding the application of HUC in regenerative medicine, we explain details of the HUC structure and its use as a patch, either fresh or cryopreserved. HUC byproducts such as MSCs and extracts have a great potential for clinical application; we describe its characteristics and regenerative properties. In addition, we discuss the potential future applications of HUC and its byproducts. Precise identification of the regenerative properties might lead to specific biosynthesis and pharmaceutical production (Iftimia-Mander et al., 2013). Additionally, we contribute with original data regarding HUC byproducts application *in vitro* and we propose that different extraction methods might produce differences in healing outcome.

## Previous Relevant Work

Human umbilical cord can be easily collected after birth and applied fresh, using the newborn’s own, to treat tissue defects in autologous procedures (Živković, 1991; Sandler et al., 2004; Álvaro and Edwin, 2017). It can also be processed in order to obtain byproducts that are used in allogenic therapies (Dardik et al., 1976; Živković, 1991; Iftimia-Mander et al., 2013; Caputo et al., 2016; Wilson et al., 2019). Since the 1970s, it has been reported that HUC and its byproducts have great potential in the repair and regeneration of injured tissues. Irving and Herbert Dardick used umbilical cord segments as skin grafts for regeneration and patented the procedure (Dardik and Dardik, 1976). They also transplanted HUC vein allografts for vascular lesion repair. HUC is easy to handle, and was noted to be highly biocompatible and resistant to biological degradation in the treated patients (Dardik and Dardik, 1976; Dardik et al., 1976).

The initial reports of HUC use in pediatric surgery were published by Heaton, Samii and Jafroudi, Liu, and Komuro, who successfully used it as a patch to treat gastroschisis (Heaton et al., 1970; Samii and Jafroudi, 1974; Živković, 1991; Komuro et al., 1998; Liu et al., 2018). The use of HUC as a patch in gastroschisis included therapeutic options such as the silon pouch procedure and the use of skin flaps or other techniques to cover the wound (Živković, 1991). The use of fresh or cryopreserved HUC patch was tested to repair spina bifida defects in preclinical settings with positive results (Papanna et al., 2015b, 2016a,b).

Additionally, HUC Blood (HUCB) has shown therapeutic effects in hematopoietic disorders and in cancer treatment (Iftimia-Mander et al., 2013; Roura et al., 2015). In 1989, HUC blood was demonstrated to have Hematopoietic Stem Cells (HUC-HSCs) with the potential to be used for bone marrow transplants (Broxmeyer et al., 1989). Nowadays, HUC blood has been approved by the U.S. Food and Drug Administration (FDA) to treat diseases such as leukemia, lymphomas, sickle cell disease and Wiskott–Aldrich syndrome (Food and Drug Administration, 2019). HUC blood HSCs have functional differences with their bone marrow counterparts as they have a greater proliferation and expansion potential and are less immunogenic, so are more suitable for therapy (Broxmeyer et al., 1989; Mayani and Lansdorp, 1998; Smythe et al., 2007; Tiwari et al., 2016). HUC-HSCs intravenous infusion of banked autologous and matched sibling treatment for certain neurological conditions has demonstrated safety and feasibility in clinical trials (phase I/II) (McLaughlin et al., 2019). Furthermore, the observed results provided the bases for using HUC-HSCs in an Expanded Access Program for acquired neurological conditions. In this, 276 children received 302 HUC blood infusions; transient infusion reactions were reported in 3.9% of the cases (*n* = 12, one reported serious adverse event), while no infections were observed and varied levels of improvement were reported. So far, these results imply that standard clinical trials phase II/III are necessary to evaluate efficacy (McLaughlin et al., 2019).

Human umbilical cord, compared to other tissues such as bone marrow or fat, is a better source of mesenchymal stem/stromal cells (MSCs), as it is easy to access and readily available in great quantities after birth (Can and Karahuseyinoglu, 2007; Wu et al., 2018). Couto et al. (2019) discussed that of 178 HUC-MSCs reported clinical trials, 20% were published. Of these, 18% concluded that MSCs were safe to use, and in 74% of the publications some form of improvement was evidenced. Moreover, a representative number of publications did not report essential aspects of the isolation method and culture (Couto et al., 2019). This lack of information challenges consensus and agreements on procedures and key technical features that are crucial for the reproducibility in methodology, and that might influence the therapeutic effect. Applications of HUC-MSCs will depend on rigorous quality controls (Wilson et al., 2019) and published evidence of positive effects of HUC-MSCs; future use in more clinical applications is foreseen.

Human umbilical cord contains factors that stimulate cell proliferation, migration, tissue differentiation, and growth (Lago et al., 2005; Secunda et al., 2015; Bruschi et al., 2018). Previous work has shown that HUC components can be isolated and used as decellularized scaffolds or extracts which have great regenerative potential (Bakhtyar et al., 2017). Decellularized WJ (DWJ) from HUC was tested as a scaffold where cells migrate and proliferate *in vitro*. As an example, HUC DWJ was used in a murine calvarial defect, where osteocytes migrated and promoted tissue reconstruction (Jadalannagari et al., 2017). DWJ, as an ingredient for a biological matrix, has shown to be an effective scaffold for HSC culture, preserving their differentiation potential and inducing the CXCR4 expression in UCB CD34+ cells, improving its transmigration abilities and providing opportunities for future applications in clinical settings (Li et al., 2019). It was in 1947 that Hadidian and Pirie prepared an extract of HUC to obtain hyaluronic acid. Since then, the HUC extract, has been used in lotions and creams for the treatment of skin diseases (Lago et al., 2005). It has also been reported that HUC WJ extract improved the regenerative properties of bone marrow MSCs in experimental models of osteoporosis (Saito et al., 2018). In conclusion, HUC decellularized scaffolds and extracts have several possible applications in regenerative medicine. However, its properties during interaction with healthy and damaged cells need further investigation (Can and Karahuseyinoglu, 2007; Papanna et al., 2015a; Mahla, 2016).

After decades of research on HUC and its byproducts, the only current clinical applications include the repair of gastroschysis and HUC blood infusion for hematopoietic disorders. A better comprehension of the effects of its byproducts by rigorous phase I, II, and III clinical trials (processed and cryopreserved HUC, isolated cells and extracts) are needed to fully understand its applications and satisfy the need for innovative and effective therapies.

## HUC Function and Structure

**FIGURE 1:**
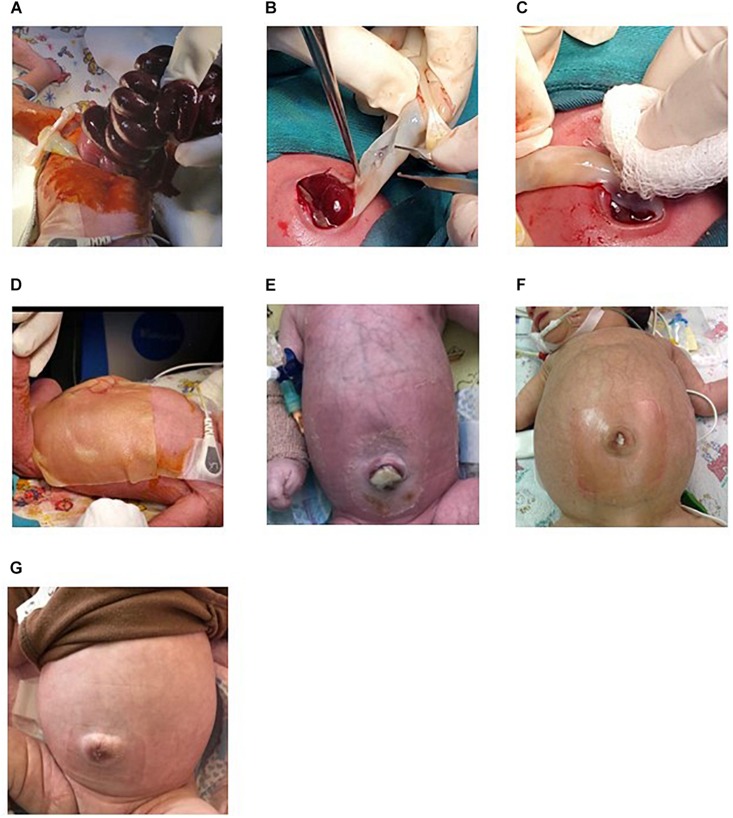
Representative images of the SGC using a HUC patch. **(A)** Manual introduction of the exposed abdominal contents. **(B)** Ligation of the HUC vessels and lengthwise division of the HUC. **(C)** Application of the exposed WJ of the HUC over the defect. **(D)** Application of the hydrocolloid patch on the defect. **(E–G)** Defect evolution at day 5, 10, and 15 after the procedure.

The HUC plays a crucial role in the development of the fetus as it supplies oxygen and nutrient-rich blood to sustain its growth. It has a unique anatomic architecture, allowing the communication between the mother and the fetus through the feto-placentary membrane and through hormones and cytokine interaction (Fahmy, 2018). The HUC develops between the 4th and 6th week of gestation (Fahmy, 2018). It has two arteries and one vein which carries oxygen and nutrient-rich blood to the fetus. The arteries and vein are embedded in a proteoglycan-rich and gelatinous WJ and surrounded by a layer of amnion (Subramanian et al., 2015; Supplementary Figure S1). The umbilical cord has to be resilient enough to assist in embryo development transporting approximately 70 liters of blood per day at a speed of 7 km/h by 31 weeks of gestation (Fahmy, 2018). By birth, the HUC has a length of about 50–60 cm, a diameter of (14.42 ± 1.50) mm and a weight of 40 g. Four compartments have been identified, the amniotic epithelial membrane (AM), the subamnion or “cord lining” (SA), the WJ and the perivascular region (PV) surrounding the umbilical blood vessels (Troyer and Weiss, 2008; Bongso and Fong, 2013; Subramanian et al., 2015; Figure 1). Each compartment has been described to contain MSCs with different characteristics. However, the role of MSCs in the HUC has not been completely elucidated; furthermore, isolating MSCs from each compartment is challenging (Subramanian et al., 2015).

During embryonic development, hematopoiesis takes place in the yolk sac and later in the aorta-gonad-mesenphoros region (AGM). Early hematopoietic and mesenchymal colonies of cells migrate from the newly formed umbilical cord toward the placenta, followed by a second round of migration from the placenta to the fetal liver and finally to the bone marrow (Wang et al., 2008). Studies have shown that these two migrations cause some MSCs to become trapped in the WJ and remain there for the duration of the gestational period (Wang et al., 2008). The jelly trapped MSCs generates sufficient microenvironment components, such as cytokines and a specific stroma, which ensure the maintenance of the cell population and the preservation of their function (Troyer and Weiss, 2008; Taghizadeh et al., 2011; Bakhtyar et al., 2017). The presence of MSCs in the HUC is the key feature of its regenerative properties (Kalaszczynska and Ferdyn, 2015; Ren et al., 2016).

## Fresh HUC as a Regenerative Patch to Treat Gastroschisis

Gastroschisis is a congenital defect, in which the abdominal wall of newborns is not fully closed (Hernández Siverio et al., 2007). The exact causes that lead to this condition are unknown (Feldkamp et al., 2007; D’Antonio et al., 2015; Schwartz and Timmapuri, 2017). It has been hypothesized that gastroschisis can be caused by a failure of the mesoderm to form the body wall, by the break of the amnion around the umbilical ring, and by an abnormal involution of the right umbilical vein, resulting in the failing of the body wall and disruption of the vitelline artery, which produce damages and herniation (D’Antonio et al., 2015), often resulting in intestinal exposure (Hernández Siverio et al., 2007). Mortality rates vary depending on the expertise of the health professionals in treating this defect, the medical center’s infrastructure and its size (Baerg et al., 2003; D’Antonio et al., 2015). In industrialized countries, 90% of treated newborns will survive. The technique used for surgical closure of abdominal wall defects in neonates is of extreme importance. Complex operations require more manipulation of the externalized organs, and sometimes a primary closure is not possible. Thus, in many occasions synthetic dressings or a preformed silo are needed (Hernández Siverio et al., 2007; Werbeck and Koltai, 2011) especially for situations where abdominal compartment syndrome is a possibility and a primary closure might be unsafe. The conventional techniques usually result in a poor cosmetic outcome (Hernández Siverio et al., 2007; Werbeck and Koltai, 2011).

Schuster and colleagues used teflon sheets to treat giant omphaloceles (Schuster, 1965). Later, Allen and Wrenn used a silastic sac, which permitted better therapeutic management of gastroschisis and omphaloceles. However, the risks of silo placement – such as infections and mechanical complications – were common and of great concern (Allen and Wrenn, 1969; Lansdale et al., 2009). Heaton et al. proposed the use of the umbilical cord as an autogenic material useful in the repair of abdominal wall defects. Later, Samii and Živkovic used the HUC as a patch to treat gastroschisis (Heaton et al., 1970; Samii and Jafroudi, 1974; Živković, 1991). This approach was later modified by Werbeck and Koltai (2011) resulting in a reduction of herniation and infections.

The HUC, and especially its WJ, is a reservoir of stem cells; specifically, WJ-MSCs might be responsible of the immunomodulatory and regenerative effects observed in gastroschisis (Ren et al., 2016). The WJ-HUC is a mucoid connective matrix with high durability and pliability, which are important features in the protection of the gastroschisis wound. The HUC patch contacts the WJ to the wound, where resident MSCs (WJ-MSCs) might induce a decrease in inflammatory reactions and wound dehiscence, decrease healing time and improve patient outcome and survival (Ferdous et al., 2018). Therefore, the results might show cell proliferation, migration, and differentiation and other regenerative effects. Nevertheless, more studies regarding the interaction between the HUC patch and the wounded tissue are needed to better understand the regenerative process.

In Hospital Carlos Andrade Marin in Quito-Ecuador, every year, 4–6 patients with gastroschisis are admitted. Dr. Edwin Ocaña routinely performs a SGC using a HUC patch. In his protocol, under general anesthesia, he performs a manual introduction of the exposed abdominal contents into the abdominal cavity, ligates the umbilical vessels, filets the umbilical cord lengthwise, without disrupting the blood vessels, and applies the exposed WJ the defect and to the reintroduced abdominal contents. He models the patch according to the size of the wall defect and applies a hydrocolloid patch on top. Local care is performed every 5 days, where the HUC is repositioned according to the extent of closure of the wall defect while covered with a new hydrocolloid (Figure 1). This procedure results in reduced healing time, decreased hospitalization, decreased duration of total parenteral nutrition and better cosmetic results (Álvaro and Edwin, 2017). These results are comparable to other reports that use a similar technique (Sandler et al., 2004).

## Applications of Preserved Cryogenic HUC

Umbilical cord patch (UCP) has shown to improve the repair of gastroschisis defects, opening the possibility to apply it in other neonatal and adult defects and wounds (Machida et al., 2011; Liu et al., 2018). Previous research showed that the cryopreservation (Papanna et al., 2015a, b) of HUC allowed its stocking and use on demand, independently of birth events. In preclinical allogeneic applications, cryopreserved HUC has shown the potential to heal myeloschisis (Papanna et al., 2015b). Scheffer Tseng and his team developed a cryopreservation technique that can prolong the amniotic membrane and the HUC’s viability, preserving its regenerative properties and opening its potential future use in human beings (Tseng et al., 2006). In Supplementary Table S1, we mention relevant protocols for HUC cryopreservation.

The cryopreserved HUC patch has been applied in chronic wounds of foot and ankle (Couture, 2016), diabetic foot ulcers (Raphael, 2016), and diabetic ulcers with osteomyelitis (Liu et al., 2008; Zhou et al., 2012; Chang and Lo, 2013; Caputo et al., 2016; Couture, 2016; Raphael, 2016; Raphael and Gonzales, 2017; Everett and Mathioudakis, 2018; Marston et al., 2019). Cryopreserved HUC effects would be due to the presence of regenerative components such as collagen and sulfated glycosaminoglycans (GAGs), hyaluronic acid, hyaluronan complexes (containing pentraxin-3), and heavy chain 1 of inter alpha inhibitor (lαl) (Tseng et al., 2012; Jadalannagari et al., 2017). Besides these current uses in research, further assays are being performed to validate its applications in others diseases such as osteoarthritis (Tseng et al., 2006). Cryopreserved HUC might be applied for purposes intended to improve healing of scars, of major abdominal surgeries (Suri and Langer, 2011) and wounds (Liu et al., 2008; Zhou et al., 2012; Chang and Lo, 2013; Papanna et al., 2015b; Caputo et al., 2016; Couture, 2016; Raphael, 2016; Raphael and Gonzales, 2017). However, more studies are needed to test these hypotheses.

## Umbilical Cord Byproducts: MSCs

In order for HUC cells to be considered MSCs, they have to comply with the criteria of the International Society for Cellular Therapy (ISCT) (Galipeau and Krampera, 2015). These criteria are as follows: adherence to treated plastic for cell culture (polystyrene), expression of CD73, CD90, and CD105; no expression of hematopoietic and endothelial markers such as HLA-DR, CD11b, CD14, CD31, CD34, and CD45; and *in vitro* differentiation potential in three lineages such as osteocytes, chondrocytes and adipocytes (Amati et al., 2018). MSCs can be found in other tissues besides HUC, such as the amniotic membrane (AM), chorionic plate (CP), decidua parietalis (DP), in the bone marrow (BM) and adipose tissue (AT), among others (Soncini et al., 2007; Chen et al., 2010; Wu et al., 2018). Regarding its properties, MSCs of the HUC, AM, and CP share a higher proliferation capacity when compared to the DP. In addition, MSCs of the HUC secrete high levels of paracrine regenerative factors such as insulin-like growth factor-1 (IGF-1) in comparison to AM and CP MSCs. Together with AM, CP and DP, HUC MSCs maintain important levels of fibroblast growth factor (FGF), human angiopoietin-1 (Ang-1), transforming growth factor beta 1 (TGF-β1), and VCAM-1 (Wu et al., 2018). HUC blood MSCs have shown a higher proliferation capacity and can be cultured for a longer period when compared to MSCs derived from the BM and AT (Kern et al., 2006).

Regenerative tests of umbilical cord byproducts are currently being assayed *in vitro*, *in vivo* and in clinical trials. MSCs from the WJ and the HUCB and decellularized extracts, have been shown to promote tissue repair and healing in patients, potentially becoming “off-the-shelf” therapies (Bartolucci et al., 2017; Riordan et al., 2018; Zhang et al., 2018). The MSCs of HUCB and of the WJ possess different characteristics. It has been shown that WJ-MSCs have better growth capacity and are easier to isolate than HUCB-MSCs (Secunda et al., 2015). However, HUCB-MSCs express lower levels of inflammatory cytokines (IL1A and IL1B) and have a high level of extracellular-matrix degradation proteins, such as MMP1 and PLAU, compared to WJ-MSCs (Doi et al., 2016). This secretory profile makes HUCB-MSCs a better therapeutic option for scarless wound healing (Doi et al., 2016). Regardless of the WJ-MSCs and HUCB-MSCs’ regenerative properties, the laboratory and manufacturing costs for their production might decrease the number of entities, public or private, that can develop therapies with these cells (Cameau et al., 2018).

Human umbilical cord-MSCs research is costly, and it is challenging for researchers and health professionals to compare the results from a variety of culture methods in order to conclude on the benefits and to obtain approval from regulatory agencies (Davies et al., 2017; Cameau et al., 2018; Ertl et al., 2018; Couto et al., 2019). The definition of MSCs requires strict criteria; however, accepted protocols for isolation, expansion and cryopreservation vary greatly (Dominici et al., 2006; Mendicino et al., 2014; Galipeau and Krampera, 2015). We propose that rigorous standardized protocols are necessary to minimize the dynamic response of MSC’s biology, heterogeneity and diversity. It is also important to emphasize that patients have significant biological variability and that their lesions are unique; those variables might also influence outcome (Clark et al., 2019; Pittenger et al., 2019). It is necessary to implement new protocols, with strict adherence to rigorousness, standardization and open access to methods.

## HUC Byproduct: Extracts

The HUC and its cells, like MSCs from WJ or HUCB, possess an active secretome [platelet-derived growth factor (PDGF), FGF, IGF-I, microvesicles (MVs) and other molecules] that have the potential to be used in regenerative applications as they stimulate cell proliferation and differentiation (Ribeiro et al., 2012; Jadalannagari et al., 2017; Kim et al., 2017; Bruschi et al., 2018). The HUC perivascular MSCs also contain anti-apoptotic agents, neuroprotective mediators against excitotoxicity, and antioxidants suitable for neural tissue regeneration (Ana et al., 2016). This cocktail can be decellularized from the raw HUC, obtaining a medium that has been tested in wound healing assays, demonstrating regenerative effects, comparable to those of the MSCs. Different isolation methods of decellularized extract have been shown to enhance wound healing *in vitro* and *in vivo* (Bakhtyar et al., 2017; Jadalannagari et al., 2017; Kočí et al., 2017).

**FIGURE 2:**
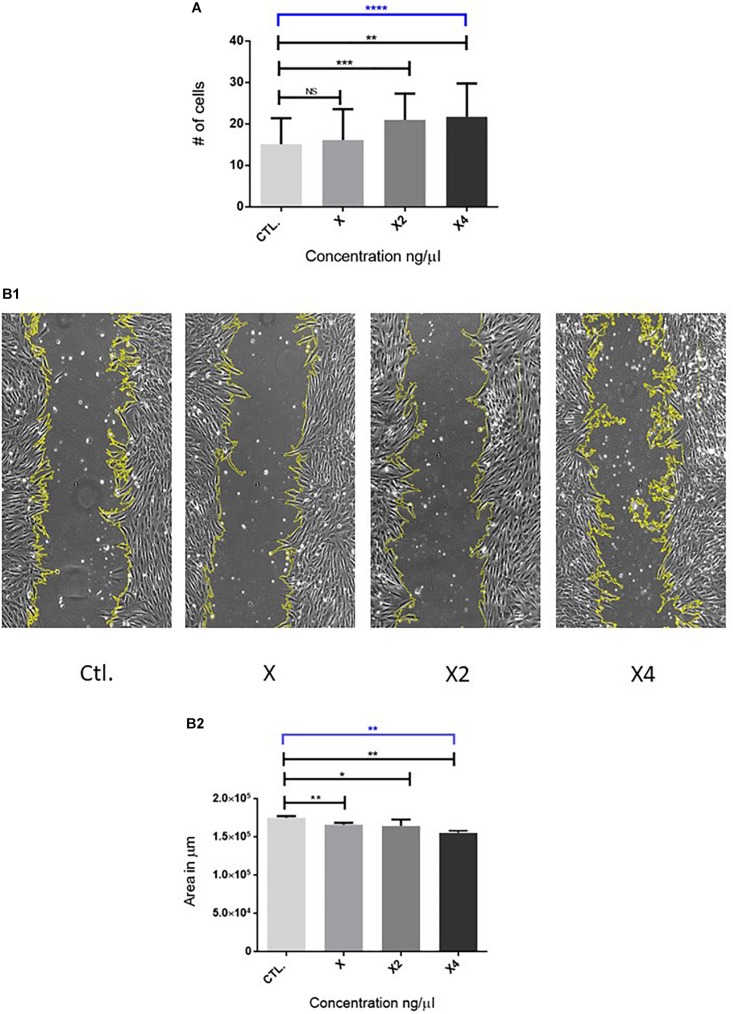
Effects of the Human Umbilical Cord (HUC) extract in proliferation, and migration of human fibroblasts (*n*=5; concerntrations were assayed in triplicates for each): **(A)**. Cell proliferation. Fibroblasts were cultured with HUC extract during 48 h. Subsequently, they were detached counted. In the graph: *X* axis coresponds to the concertration of the extract, *Y* axis represents the number of countes cells × 1000. **(B1,B2)**. Cell migration estimated with “*in-vitro* migration wound healing assay”. The area of closured was measured after 16 h HUC of extract exposure, the measurements was the wounded zones were made by using the program ImageJ. **(B1)** Representative images of the wounded area highlighted in yellow for each concertration, the length of the field is 700 μm, three fields were analyzed by well. **(B2)** Analysis of the wounded area for each concerntration. Mean and standard deviation of the wounded area measured for the concertations of the tested. The program used was GraphPad prism 6. Statistical analysis: Mean ±SD; un-paird, non-parametric, Kruskal–Wallis and Mann–Whitney post hoc tests were performed (*p<0.05, **p<0.01, ***p<0.001). The program used was GraphPad prism 6.

Human umbilical cord can be mixed, filtrated or pelleted, the extract increases the *in vitro* proliferation and migration of fibroblasts, as described in the literature (Van Pham et al., 2014; Bakhtyar et al., 2017; Nagaishi et al., 2017; Saito et al., 2018). Original data from our laboratory have shown that increasing protein concentrations of HUC extract enhance fibroblast proliferation (after 48 h) and migration (16 h) (Figure 2). In a similar study, Bakhtyar et al. (2017) observed wound healing and fibroblast migration in *in vitro* and *in vivo* murine models; no assays were performed for proliferation *in vitro* and no significant differences were reported *in vivo*. The original results from our lab were obtained using a different technique from the one described by Bakhtyar et al. (2017). Our procedure is being submitted for intellectual property protection and will be described in detail in the near future. Our results suggest that our technique could improve proliferation and migration properties of fibroblasts *in vitro* (Figure 2).

Decellularizing the HUC provides an opportunity to purify the cocktail of biochemical messengers capable of inducing regenerative effects. Our method is less expensive and more cost-effective than isolating and culturing MSCs. However, more assays need to be performed to further investigate the HUC extract, its tissue regeneration potential and immune suppressive properties (Luz-Crawford et al., 2016). Furthermore, HUC extract may serve as a foundation to produce a hydrogel matrix which has the potential to become a scaffold for neural tissue repair (Kočí et al., 2017), a 3D scaffolding material for tissue engineering (Jadalannagari et al., 2017) and provide possible applications in cosmetics (Kim et al., 2014).

## Future of HUC Extract and Its Byproducts, Identification and Biosynthesis of the Active Factors

Regenerative medicine is in continuous progress. Biochemical analysis, high throughput screening and bioinformatics contribute to advances in the area (Tomchuck et al., 2008; Arslan et al., 2013; Mitragotri et al., 2014; Akyurekli et al., 2015; Chen et al., 2016; Luz-Crawford et al., 2016; Caicedo et al., 2017; Chan et al., 2017; Ma et al., 2017; Amati et al., 2018; Court et al., 2018; de la Rosa et al., 2018; Li et al., 2018; Miliotis et al., 2019). Factors that stimulate the secretion of the biochemical cocktail, the ideal extraction techniques, active byproducts and possible combinations that lead to desired effects which respond to inflammatory signals by secreting cytokines and exosomes, need further study (Tomchuck et al., 2008; Arslan et al., 2013; Ma et al., 2017; Miliotis et al., 2019). We hypothesize that the synthesis of these factors might have potential for tissue regeneration, immune regulation and healing (Akyurekli et al., 2015; Chen et al., 2016; Caicedo et al., 2017; Court et al., 2018; Luz-Crawford et al., 2019). Continuous research and clinical trials might provide more insight on the possibility of surgical applications, wound healing and tissue repair. In addition, more analyses are needed for the identification of functionally superior subsets of cells, optimized delivery methods, scaffolding matrix, secretome activity and engraftment enhancement techniques. New technologies (Soncini et al., 2007; Chen et al., 2010; Amati et al., 2018) can be tested in the future, for the synthesis and mix of regenerative factors based on HUC byproducts. Bioinformatics might also help fine-tune the use of these byproducts, considering the genetic profile of each patient (Krauss et al., 2017). The HUC and its byproducts might set the path for the development of new therapies and their potential application in personalized regenerative medicine.

## Data Availability Statement

All data generated in this article and derived data supporting the findings of this study are available from the corresponding author on request.

## Ethics Statement

The studies involving human participants were reviewed and approved by the Comité de Ética de Investigación en Seres Humanos at the USFQ (CEISH-USFQ) and at the Hospital Carlos Andrade Marín (HCAM). Ethics Committee approval for the extract assays: Universidad San Francisco de Quito, USFQ, 2018-209IN. HCAM written informed consent to participate in this study was provided by the participants’ legal guardian/next of kin to Dr. Edwin Ocaña. Written informed consent was obtained from the minor(s)’ legal guardian/next of kin for the publication of any potentially identifiable images or data included in this article. No identifiable data or image is presented in the article.

## Author Contributions

All authors contributed for the development of this article. FV, VC, EM, MO, and AC performed the *in vitro* assays of proliferation and wound healing. EO applied the HUC treatment in the gastroschysis patients. EO, JÁ–B, AC, LE, and MG provided intellectual input and supported the development of the HUC extracts and its applications *in vitro*. LE provided the idea for investigating the HUC and its by-products, in clinical research and translational medicine. FV and AC developed the technique to obtain the HUC extract. FV, VC, EM, MO, LE, MG, and AC wrote the manuscript. LE and AC conceptualized the manuscript.

## Conflict of Interest

The authors declare that the research was conducted in the absence of any commercial or financial relationships that could be construed as a potential conflict of interest.
