# Supplementary figures and images for: Use of Human Umbilical Cord and Its Byproducts in Tissue Regeneration
Source: Front Bioeng Biotechnol. 2020 Mar 10;8:117. doi: 10.3389/fbioe.2020.00117 (PMC7075856; doi:10.3389/fbioe.2020.00117)

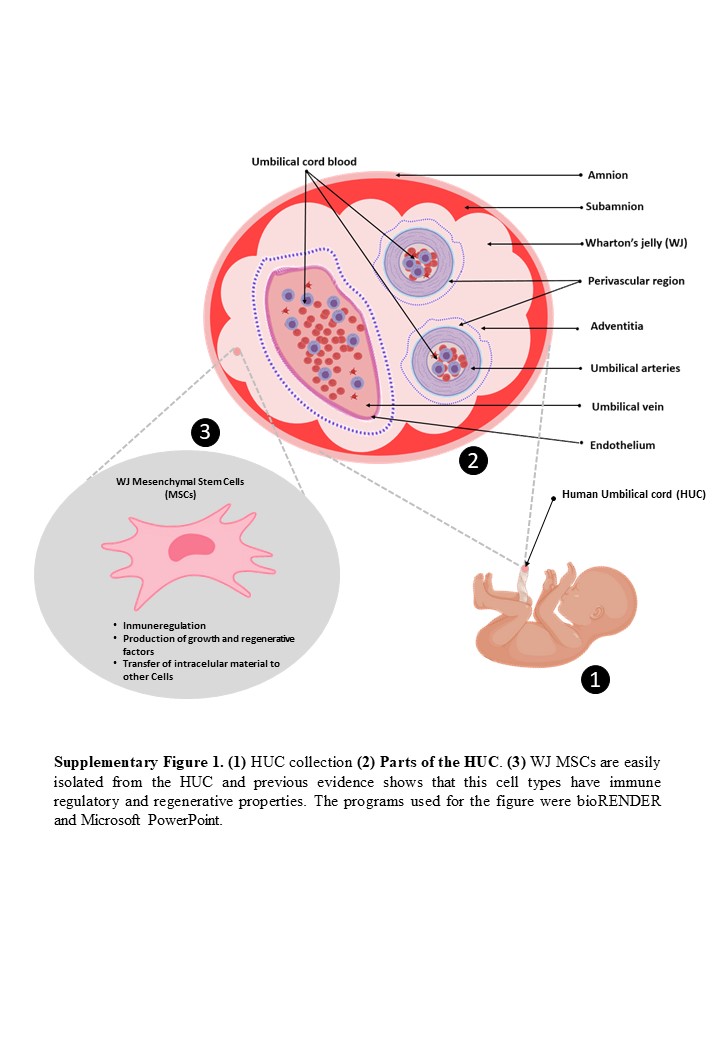

Supplement: Supplementary file 1 [file Image_1.JPEG]
