# Supplementary material for: Use of Human Umbilical Cord and Its Byproducts in Tissue Regeneration
Source: Front Bioeng Biotechnol. 2020 Mar 10;8:117. doi: 10.3389/fbioe.2020.00117 (PMC7075856; doi:10.3389/fbioe.2020.00117)
Supplement: Supplementary file 2 [file Table_1.DOCX]

Supplementary Table 1: Current methods and procedures of HUC Wharton’s Jelly cryopreservation.

| **Tissue Preparation**  **(obtaining, washing and fragmentation)** | **Preservation** | **Reference** |
| --- | --- | --- |
| Blood is squeezed from the HUC. Tissue is sterilized using Okteniderm Antiseptic. HUC is cut into fragments (~2×2×2 mm) and centrifuged. The tissue fragments are washed extensively with sterile saline solution and transferred to cryotubes. 3 ml of cold MSC culture medium with 10% serum and 10% DMSO are added for cryopreservation. | Standard procedures are used according to Cord Blood Bank guidelines. Hematopoietic stem cells from the umbilical cord blood are frozen to -90°C. Tubes are transferred to a quarantine Dewar flask and stored in liquid nitrogen vapor at -160°C. | Romanov, Y. A., Balashova, E. E., Volgina, N. E., Kabaeva, N. V., Dugina, T. N., & Sukhikh, G. T. (2016). Isolation of Multipotent Mesenchymal Stromal Cells from Cryopreserved Human Umbilical Cord Tissue. Bulletin of Experimental Biology and Medicine, 160, 530-534. |
| PBS (Phosphate buffered saline) is used to wash cord tissue. The tissue is cut into fragments (0.5-1 cm). Blood vessels are removed from the cord tissue fragments. The remaining Wharton’s jelly is divided into 1-2-mm^3^ fragments. The tissue fragments are chopped and mixed with 10% DMSO in heat –inactivated autologous cord plasma. | The small fragments are transferred into a 5-mL VueLife bag or to cryovials and stored at -80°C. | Friedman, R., Betancur, M., Boissel, L., Tuncer, H., Cetrulo, C., & Klingemann, H. (2007). Umbilical Cord Mesenchymal Stem Cells: Adjuvants for Human Cell Transplantation. Biology of Blood and Marrow Transplantation, 13, 1477-1486. |
| PBS is used to wash the HUC and remove blood clots. The tissue is frozen at -80°C.The HUC is thawed at room temperature for 8 hours according to Good Manufacturing Practice (GMP) guidelines. HUC is placed at 8°C for 16 hours. Blood vessels are removed from the HUC, and PBS is again used to remove possible remaining blood. Cord tissue is cut into fragments. | HUC tissue fragments are placed in a container with 1:1 v/v Dulbecco Modified Eagle Medium and glycerol, before being stored at -80°C for a maximum two years. | Cooke, M., Tan, E., Mandrycky, C., He, H., O'Connell, J., & Tseng, S. (2014). Comparison of cryopreserved amniotic membrane and umbilical cord tissue with dehydrated amniotic membrane/chorion tissue. Journal of Wound Care, 23(10). |
| The HUC is chopped into 2-3 cm segments and is washed with saline buffer to eliminate blood and blood clots. | Cord fragments are stored in normal saline containing 1X Antibiotic-Antimycotic at 4°C for 2 hours. 10% DMSO + 0.2 M sucrose is added to the cord fragments. Frozen overnight in cryotubes reducing temperature at a rate of 1°C/ minute, from room temperature to -80°C. HUC fragments are transferred to liquid nitrogen and stored at -196°C for 5-6 days. | Roy, S., Arora, S., Kumari, P., & Ta, M. (2014). A simple and serum-free protocol for cryopreservation of human umbilical cord as source of Wharton’s jelly mesenchymal stem cells. Cryobiology, 68(3), 467-472. |
| HUC donors’ blood is tested for different infectious diseases and they have to come up negative. HUC is obtained immediately after delivery of a healthy baby, it is washed several times with balanced salt saline, which contains antibiotics. The HUC is cut into small pieces of different sizes (between 1.2 and 3 cm). | Microbiological tests are done to ensure no microbial nor fungal growth in the tissue. The cell activity in the tissue is made inactive with the help of CryoTek^®^ (cryopreservation method): The tissue is stored in DMEM medium 1:1 (v/v) with Glycerol. with 20 µg/ml Ciprofloxacin and 1.25 µg/ml Amphotericin B. Packaging is placed at -80°C until needed. Thus, after freezing the tissue pieces, the remaining membrane’s cells are killed and therefore the graft is not rejected by the patient. | Papanna, R. *et al.* Cryopreserved human umbilical cord patch for in-utero spina bifida repair. *Ultrasound Obstet. Gynecol.* **47**, 168–176 (2015).  Caputo, W. J. *et al.* A retrospective study of cryopreserved umbilical cord as an adjunctive therapy to promote the healing of chronic, complex foot ulcers with underlying osteomyelitis. *Wound Repair Regen. Off. Publ. Wound Heal. Soc. Eur. Tissue Repair Soc.* **24**, 885–893 (2016).  Papanna, R., Fletcher, S., Moise, K. J., Mann, L. K. & Tseng, S. C. G. Cryopreserved Human Umbilical Cord for In Utero Myeloschisis Repair (2016).  Papanna, R. *et al.* Neurological Outcomes after Human Umbilical Cord Patch for In Utero Spina Bifida Repair in a Sheep Model. *AJP Rep.* **6**, e309-317 (2016).  Couture, M. A Single-center, Retrospective Study of Cryopreserved Umbilical Cord for Wound Healing in Patients Suffering From Chronic Wounds of the Foot and Ankle. *Wounds Compend. Clin. Res. Pract.* **28**, 217–225 (2016).  Raphael, A. A single-centre, retrospective study of cryopreserved umbilical cord/amniotic membrane tissue for the treatment of diabetic foot ulcers. *J. Wound Care* **25**, S10–S17 (2016).  Raphael, A. & Gonzales, J. Use of cryopreserved umbilical cord with negative pressure wound therapy for complex diabetic ulcers with osteomyelitis. *J. Wound Care* **26**, S38–S44 (2017).  Marston, W. A. *et al.* An open-label trial of cryopreserved human umbilical cord in the treatment of complex diabetic foot ulcers complicated by osteomyelitis. *Wound Repair Regen.* **27**, 680–686 (2019). |
| The umbilical cord is obtained, cleaned with alcohol 70-75% and PBS. It is chopped into pieces of maximum 2 mm. Enzyme treatment can be used (trypsin / collagenase / dispase / gelatase / hyaluronidase). To stop enzyme action, human serum, a solution with EDTA or FCS is added. The umbilical cord pieces are washed with PBS. | The umbilical cord pieces are mixed with a cryogenic composition (DMEM / PBS and human cord blood serum / FCS and dextran / sucrose / trehalose); a cryoprotectant (DMSO / glycerol / ethylene glycol / propylene glycol) and a protein (albumin / PPF). The mixture is shaken for 28-32 minutes at a temperature >15° C., or a temperature of 0°C-10°C., or at a temperature of maximum 4°C. The mixture is then cryopreserved at -196°C. | Chang, H. K., & Lo, W. Y. (2013). *U.S. Patent No. 2013/0059286 A1*. |
